# Cardiopulmonary Bypass and Cross-Clamping Times in Aortic Valve Replacement Surgery by Ministernotomy with Sutureless Prosthesis Implantation Compared to Conventional Prosthesis: A Cross-Sectional Study

**DOI:** 10.21470/1678-9741-2024-0290

**Published:** 2025-03-13

**Authors:** Álvaro Perazzo, Silvia Mariani, Gabriela Lucena Montenegro, Luca Conci, Diana Patrícia Lamprea Sepúlveda, Samuel Padovani Steffen, Fabio Antonio Gaiotto, Roberto Lorusso, Silvio Caldas Neto, Esdras Marques Lins, João Carlos Ferreira Leal, Fernando Ribeiro de Moraes Neto

**Affiliations:** 1 Department of Surgery, Postgraduate Program in Surgery, Centro de Ciências Médicas, Universidade Federal de Pernambuco (UFPE), Recife, Pernambuco, Brazil; 2 Transplant Center, Instituto do Coração, Faculdade de Medicina, Universidade de São Paulo (USP), São Paulo, São Paulo, Brazil; 3 Cardio-Thoracic Surgery Department, Maastricht University Medical Center+ and Cardiovascular Research Institute Maastricht (CARIM), Maastricht, The Netherlands; 4 Cardiac Surgery Unit, Cardiothoracic and Vascular Department, Fondazione IRCCS San Gerardo dei Tintori, Monza, Italy; 5 Grupo de Intervenções Coronarianas e Estruturais do Coração (ICECOR), Recife, Pernambuco, Brazil; 6 Postgraduate Program in Translational Medicine, Universidade Federal de São Paulo (UNIFESP), São Paulo, São Paulo, Brazil; 7 Pronto-Socorro Cardiológico de Pernambuco (PROCAPE), Universidade de Pernambuco, Recife, Pernambuco, Brazil; 8 Department of Cardiac and Thoracic Aortic Surgery, Medical University of Vienna, Vienna, Austria; 9 Instituto do Coração de Pernambuco (InCor-PE), Real Hospital Português, Recife, Pernambuco, Brazil; 10 Department of Surgery, Faculdade de Medicina de São José do Rio Preto (FAMERP), São José do Rio Preto, São Paulo, Brazil; 11 Instituto D’Or de Pesquisa e Ensino (IDOR), Recife, Pernambuco, Brazil

**Keywords:** Aortic Valve, Bioprosthesis, Cardiopulmonary Bypass, Constriction, Left Ventricular Function, Aortic Valve Disease, Ischemia

## Abstract

**Introduction:**

Valve replacement is one of the effective treatments for aortic valve disease. This study aims to compare cardiopulmonary bypass and ischemia times in aortic valve replacement surgeries using stented biological and sutureless prostheses (PERCEVAL^®^) through a minimally invasive ministernotomy approach.

**Methods:**

This single-center cross-sectional study, conducted from February 2015 to February 2021, assessed clinical and epidemiological characteristics in aortic valve replacement patients. It analyzed factors including hospital stay, early outcomes, valve etiology, intraoperative diagnosis, systolic gradients, left ventricular ejection fraction, and left ventricular mass. Two groups were studied: 12 patients with PERCEVAL^®^ prostheses and 81 with conventional bioprostheses.

**Results:**

This study included 93 patients (age: 59 ± 16 years), 61.3% were male, and 80.2% had hypertension; dyslipidemias were present in 34.1% and 25.3% were diabetic. Cardiopulmonary bypass and cross-clamping times were 61 minutes and 41 minutes in the conventional bioprostheses group and 59.5 minutes and 39.5 minutes in the PERCEVAL^®^ group (*P*=0.143 and *P*=0.058, respectively). Intensive care unit and overall hospital stays were statistically comparable between both groups (*P*=0.662 and *P*=0.599, respectively). All participants survived the 30-day postoperative period, with minimal complications, no significant differences in echocardiographic parameters were observed, yet higher values for certain cardiac function indicators were noted in the conventional bioprostheses group.

**Conclusion:**

The groups with conventional bioprostheses and sutureless prostheses (PERCEVAL^®^) didn't display significant differences in the analyzed variables for ministernotomy aortic valve replacement surgery. They exhibited similar results in terms of hospital stay duration, 30-day outcomes, and cardiac function values.

## INTRODUCTION

The prevalence of cardiovascular diseases has seen a notable rise, currently accounting for approximately 32% of global causes of death, with over 400,000 deaths occurring annually in Brazil^[1,2]^. This increase has been attributed to factors such as elevated life expectancy and changes in dietary habits. Among these diseases, aortic valve diseases, particularly aortic stenosis, have been showing an increasing incidence, especially in industrialized countries^[3,4,5]^. Degenerative aortic stenosis, prevalent in elderly individuals, affects around 6% of the population over 65 years, emerging as a significant issue in Brazil. It is estimated that, by 2030, more than 900,000 individuals will require surgical intervention due to aortic valve disease in Brazil^[3,6]^. The standard treatment for symptomatic cases of aortic stenosis includes surgical valve replacement through minimally invasive procedures, such as ministernotomy, which are gaining prominence for promoting quicker recovery and reducing costs^[7,8]^.

In this context, the PERCEVAL® sutureless prosthesis emerges as an innovative alternative to conventional options. This prosthesis stands out for reducing cardiopulmonary bypass (CPB) and aortic cross-clamping times, enhancing clinical outcomes, and offering advantages such as easy implantation, adaptability to patient anatomy, and predictable results^[9,10,11]^. Its hemodynamic performance is superior to other prostheses and is comparable to transcatheter aortic valve implantation^[10,11,12]^.

However, despite advancements, pre and intraoperative risk factors still significantly impact the morbidity and mortality rates of patients undergoing cardiac surgery. It is essential to evaluate whether approaches like ministernotomy and the use of sutureless prostheses can minimize these risks^[13,14,15]^. Hence, this study aims to evaluate and compare the CPB and aortic cross-clamping times in aortic valve replacement surgery through ministernotomy, using the PERCEVAL® sutureless prosthesis and the conventional ones. Additionally, it aims to characterize the epidemiological and clinical profile of patients, to analyze pre and postoperative echocardiographic parameters, and to assess the impact of CPB and cross-clamping times on clinical variables and hospital outcomes. This study seeks to contribute to optimizing therapeutic approaches in aortic stenosis and to provide valuable insights for clinical practice and medical decision-making.

## METHODS

### Study Design

This study is an observational, retrospective, cross-sectional single-4centre study.

### Patient Selection

The medical records of patients who underwent aortic valve replacement surgery through ministernotomy, with the implantation of conventional aortic valve prosthesis and PERCEVAL® (sutureless prosthesis), were analyzed from February 2015 to February 2021. The CPB and aortic cross-clamping times were recorded. All surgeries were performed by the team at the Instituto do Coração de Pernambuco (InCor-PE). Additionally, postoperative status was evaluated through transthoracic echocardiography. The patients were divided into two groups:

- Patients who received PERCEVAL® prostheses.
- Patients who received conventional prostheses.

Informed Consent Forms (ICFs) were obtained for willing participants, while for unreachable participants, ICFs were requested from the family/responsible party, with justification for its waiver when contact was not possible, as detailed in the “Ethical Aspects” section. The primary objectives were to evaluate and compare CPB and aortic cross-clamping times. The secondary objectives were the evaluation and comparison of pre and postoperative echocardiograms.

### Eligibility

#### Inclusion Criteria

- Patients aged over 18 years, who underwent elective isolated aortic valve replacement surgery by ministernotomy.
- Severe aortic valve stenosis, defined by transvalvular aortic gradient > 40 mmHg, jet velocity > 4 m/s, or a valvular orifice < 1.0 cm^2^.
- Or dual aortic lesion, defined as the presence of predominant severe aortic stenosis with mild or moderate valvular regurgitation.
- Sinotubular junction diameter > 24.7 mm and < 35.1 mm, and annulus diameter > 19 mm and < 27 mm.
- Sinotubular junction to annulus diameter ratio < 1.3, for the PERCEVAL® group.
- Symptomatic patients due to valvular stenosis, with New York Heart Association (NYHA) functional class > II.

#### Exclusion Criteria

- Multivalvular disease.
- Annular diameter < 18 mm or > 28 mm for the conventional bioprostheses group.
- Associated procedures.
- Sternotomy.

### Study Location

The study was based on the analysis of the medical records of patients operated on at the InCor-PE, as well as at the Real Hospital Português and at the D’Or/São Luiz Network Hospitals (Hospital Esperança Olinda, Hospital Memorial São José, and Hospital São Marcos), all located in Recife, Pernambuco, Brazil.

### Technical Procedures

#### Preoperative Assessment

A multidisciplinary group (Heart Team) was responsible for evaluating, diagnosing, and indicating patients for surgery, after clinical, laboratory, echocardiographic, and ultrasonographic examinations. These selected patients, in addition to clinical symptoms, met the formal indications outlined in the most current guidelines^[16,17]^. All patients underwent transthoracic echocardiography to assess morphological and functional aspects of the left ventricle, aortic valve diameter and area, and maximum and medium systolic gradients.

#### Intraoperative Assessment

CPB time is measured from the initiation of CPB, where mechanical circulatory support begins, until its cessation. The cross-clamping time is counted from the moment when the aorta is clamped until the release of the aorta and myocardial reperfusion.

#### Postoperative Assessment

Postoperative complications such as stroke, myocardial infarction (MI), excessive bleeding, total atrioventricular block, need for a permanent pacemaker, acute renal failure requiring dialysis, respiratory failure requiring reintubation or tracheostomy, and infectious complications such as mediastinitis and sternal dehiscence were monitored. Transthoracic echocardiography was performed after the immediate postoperative period to assess left ventricular ejection fraction, prosthetic valve position, systolic gradients, valvar area, and left ventricular mass providing a comprehensive evaluation of cardiac function.

### Statistical Analysis

Data were collected and analyzed with Microsoft Excel® and IBM Corp. Released 2011, IBM SPSS Statistics for Windows, version 20.0, Armonk, NY: IBM Corp. software for statistical analysis.

Continuous variables were presented as mean ± standard deviation. Categorical variables were described as absolute (n) and relative (%) frequencies.

Comparisons between patients who received the PERCEVAL® prosthesis and those who received the conventional prosthesis were performed with chi-square test or Fisher’s exact test for categorical variables and Mann-Whitney U test for continuous variables.

The parameters of transthoracic and transesophageal echocardiography in the pre and postoperative periods were descriptively analyzed using median along with minimum and maximum values. To check for differences in these parameters before and after the surgical procedure, the Wilcoxon test for paired samples was utilized.

Differences were considered significant at a level of 5% (*P*-value ≤ 0.05).

### Ethical Aspects

Ethical principles outlined in the Declaration of Helsinki were adhered to throughout the research process, from conceptualization to dissemination of knowledge and its application in professional practice. The research project received approval from the Research Ethics Committee of the Universidade Federal de Pernambuco, under the Certificado de Apresentação e Apreciação Ética (CAAE) number 43279321.8.1001.5208. It also received approval from the research core of the Real Hospital Português de Beneficência in Pernambuco, the Research Support Core of the Instituto D'Or de Pesquisa e Ensino (NAPE/IDOR-Recife-Pernambuco), and the Human Research Ethics Committee of the Instituto de Medicina Integral Prof. Fernando Figueira (CAAE number 43279321.8.2001.5201). These approvals were granted in accordance with the protocols submitted by Plataforma Brasil. The sutureless prosthesis (PERCEVAL®) and conventional prostheses used in this study are approved by the Agência Nacional de Vigilância Sanitária (or ANVISA) and are routinely used and commercially available in Brazil.

Ethical aspects were observed in accordance with Resolution no. 466/2012 of the Brazilian Health Council (Conselho Nacional de Saúde/Comissão Nacional de Ética em Pesquisa). Consequently, the application of the ICF was anticipated. In cases of unsuccessful contact with the patient or communication impediments, attempts were made to contact family members or guardians, and if unsuccessful, a waiver of ICF was applied.

## RESULTS

### Clinical Data

**Fig. 1:**
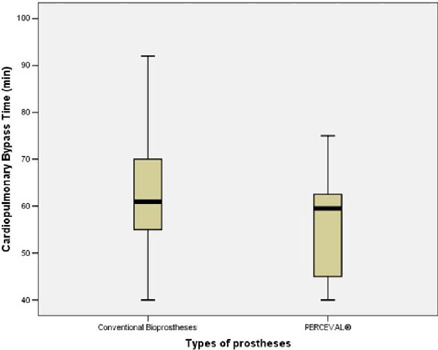
Cardiopulmonary bypass time for conventional bioprostheses and PERCEVAL®.

**Fig. 2:**
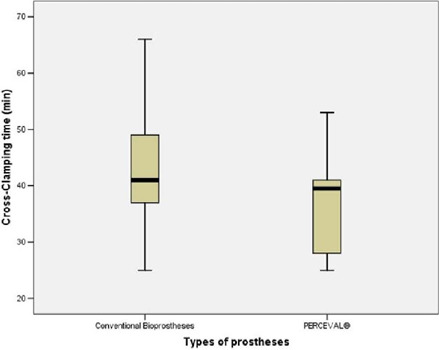
Cross-clamping time for conventional bioprostheses and PERCEVAL®.

The epidemiological and clinical profile of all operated patients is displayed in Table 1. We had 93 patients for all population, where 81 were in the conventional bioprostheses group and 12 were in the PERCEVAL® group. Predominantly, the study participants were male (n=61.3%), with a median age of 61 years, median weight of 77 kilograms, and median height of approximately 165 centimeters. Patients who received conventional bioprostheses were younger (median age: 60 years, range: 44-69 years) compared to those implanted with the PERCEVAL® prosthesis (median age: 76.5 years, range: 60.8-79.8 years) (*P*=0.003). A significant proportion of patients had hypertension (80.2%), with 25% having diabetes, 12% being smokers, 3% reporting alcohol use, and 20% classified as obese. Regarding the clinical profile, 20.9% exhibited coronary artery disease (CAD), and only 4.4% had experienced a stroke. Among all patients, 14.3% had rheumatic conditions, 3.3% had aortic valve endocarditis, and 78% showed valve calcification. Patients who received conventional bioprostheses did not show longer CPB and cross-clamping times (Table 2 and Figures 1 and 2). Overall, the median intensive care unit stay was two days (range: 1-4 days), and the overall hospital stay was eight days (range: 7-13) with no significant differences between the types of prostheses (Table 3). Furthermore, no deaths, MI, or postoperative tamponade were recorded in the early 30-day outcomes. Also, regarding the etiology of the valve disease, no patients with myxomatous/ degenerative origin of the aortic valve were identified. Bleeding was experienced in 2% of patients, 4.4% underwent permanent pacemaker implantation, and 1.1% incurred a stroke shortly after the surgery.

**Table 1:** Preoperative characteristics.

Type of aortic valve prosthesis							
	Conventional bioprosthesis (n = 81)		PERCEVAL® (n = 12)		Total		*P*-value**
	n	%	n	%	n	%	
Age (years)*	60.0 (44.0; 69.0)		76.5 (60.8; 79.8)		61.0 (46.0; 73.0)		0.003***
Sex							
Male	52	64.2%	5	41.7%	57	61.3%	
Female	29	35.8%	7	58.3%	36	38.7%	0.203
Weight (kg)*	78.0 (66.3; 91.5)		69.0 (64.0; 79.0)		77.0 (65.0; 88.0)		0.145***
Height (cm)*	167 (162; 172)		158 (153; 160)		165.5 (158.8; 172,0)		0.009***
Systemic arterial hypertension	62	78.5%	11	91.7%	73	80.2%	0.448
Diabetes mellitus	18	22.8%	5	41.7%	23	25.3%	0.171
Smoking	10	12.7%	1	8.3%	11	12.1%	1.000
Alcoholism	3	3.8%	0	0.0%	3	3.3%	1.000
Chronic kidney disease	2	2.5%	1	8.3%	3	3.3%	0.349
Dyslipidemia	25	31.6%	6	50.0%	31	34.1%	0.326
Obesity	14	17.7%	4	33.3%	18	19.8%	0.245
Atrial fibrillation	5	6.3%	1	8.3%	6	6.6%	0.583
Arrhythmia	9	11.4%	2	16.7%	11	12.1%	0.635
Stroke or cerebrovascular accident	4	5.1%	0	0.0%	4	4.4%	1.000
Coronary artery disease	14	17.7%	5	41.7%	19	20.9%	0.119

*Results presented as median (minimum; maximum),

**Chi-square test (or Fisher's exact test, when necessary),

***Mann-Whitney U test

**Table 2:** Cardiopulmonary bypass and cross-clamping times.

	Type of aortic valve prosthesis		Total	*P*-value**
	Conventional bioprosthesis (n = 81)	PERCEVAL® (n = 12)		
Cardiopulmonary bypass time (min)*	61.0 (55.0; 70.0)	59.5 (45.0; 62.8)	60.0 (53.5; 69.5)	0.143
Cross-clamping time (min)*	41.0 (37.0; 49.0)	39.5 (28.0; 41.5)	40.0 (36.0; 47.5)	0.058

*Data presented in the form of median (Q1; Q3),

**Mann-Whitney U test

**Table 3:** Intra and postoperative outcomes.

	Type of aortic valve prosthesis				Total		*P*-value*
	Conventional bioprosthesis (n = 81)		PERCEVAL® (n = 12)				
	n	%	n	%	n	%	
**Length of stay**							
Length of stay in the cardiothoracic recovery unit in days, median (Q1; Q3)	2 (1; 4)		2 (1; 3.8)		2 (1; 4)		0.662**
Length of hospital stay in days, median (Q1; Q3)	8 (7; 13)		11 (6.3;13.8)		8 (7; 13)		0.599**
**Early outcome in 30 days**							
Early postoperative bleeding	2	2.5%	0	0.0%	2	2.2%	1.000
Early postoperative permanent pacemaker implantation	4	5.1%	0	0.0%	4	4.4%	1
Early postoperative cerebrovascular accident	1	1.3%	0	0.0%	1	1.1%	1.000
**Etiology of valve disease**							
Valve disease of rheumatic etiology	13	16.5%	0	0.0%	13	14.3%	0.203
Aortic valve endocarditis involvement	3	3.8%	0	0.0%	3	3.3%	1.000
Aortic valve calcification	59	74.7%	12	100.0%	71	78.0%	0.062
**Intraoperative diagnosis**							
Aortic regurgitation							
Mild	26	32.9%	4	33.3%	30	33.0%	
Moderate	32	40.5%	8	66.7%	40	44.0%	
Severe	21	26.6%	0	0.0%	21	23.1%	0.056
Aortic valve stenosis							
Mild	4	5.1%	0	0.0%	4	4.4%	
Moderate	19	24.1%	0	0.0%	19	20.9%	
Severe	56	70.9%	12	100.0%	68	74.7%	0.133
Double valve injury							
Aortic stenosis > aortic regurgitation	33	41.8%	9	75.0%	42	46.2%	
Aortic regurgitation > aortic stenosis	1	1.3%	0	0.0%	1	1.1%	
Aortic regurgitation = aortic stenosis	2	2.5%	0	0.0%	2	2.2%
No	43	54.4%	3	25.0%	46	50.5%	0.179
Bioprosthetic aortic valve dysfunction							
Yes	0	0	0	0	0	0	
No	79	100.0%	12	100.0%	91	100.0%	-
Mechanical prosthetic aortic valve dysfunction							
Yes	0	0	0	0	0	0
No	79	100.0%	12	100.0%	91	100.0%	-

*Chi-square test (or Fisher’s exact test, when necessary),

**Mann-Whitney U test

### Echocardiographic Data

Comparing the pre and postoperative echocardiographic values for all patients submitted to the aortic valve surgery, statistically significant differences emerge (Table 4). These differences encompass the left ventricular ejection fraction (*P*=0.010), diastolic diameter of the left ventricle (*P*=0.002), left ventricular mass (*P*=0.003), maximum systolic gradient (*P*<0.001), and mean systolic gradient (*P*<0.001). Noteworthy, higher median values were identified in the pre-surgical period.

**Table 4:** Transthoracic echocardiography in the pre and postoperative periods for all patients.

Variable*	n	Preoperative	n	Postoperative	P-value**
**Transesophageal echocardiography**					
Left ventricular ejection fraction (%)	4	62 (52; 66)	4	63 (45; 78)	0.317
Left ventricular systolic diameter (mm)	3	44 (36; 53)	2	35.5 (34; 37)	-
Left ventricular diastolic diameter (mm)	3	66 (57; 73)	2	49.5 (44; 45)	-
Interventricular septum (mm)	3	10.0 (9.0; 13.0)	2	11.5 (11.0; 12.0)	-
Posterior wall (mm)	3	10.0 (8.0; 11.0)	2	11.5 (11.0; 12.0)	-
Left ventricular mass (g)	3	291.0 (261.2; 360.1)	3	178.0 (171.0; 273.1)	0.317
Maximum systolic gradient (mmHg)	4	65.0 (36.0; 77.7)	4	50.5 (18.0; 68.8)	-
Mean systolic gradient (mmHg)	1	49.1 (49.1; 49.1)	4	30.5 (11.0; 38.0)	-
**Transthoracic echocardiography**					
Left ventricular ejection fraction (%)	72	65 (40; 78)	47	61 (50; 76)	0.010
Left ventricular systolic diameter (mm)	60	34 (23; 59)	26	34.5 (23; 59)	0.018
Left ventricular diastolic diameter (mm)	59	54.0 (38.0; 166.6)	26	50 (38; 81)	0.002
Interventricular septum (mm)	55	12 (7; 15)	26	11 (8; 17)	0.824
Posterior wall (mm)	49	11 (8; 15)	26	11 (8; 16)	0.943
Left ventricular mass (g)	48	237.0 (90.0; 575.9)	38	204.0 (107.0; 549.4)	0.003
Maximum systolic gradient (mmHg)	61	79 (14; 138)	40	24.0 (6.8; 116.0)	< 0.001
Mean systolic gradient (mmHg)	60	48.5 (6.0; 138.0)	38	15 (2; 64)	< 0.001

*Results presented as median (minimum; maximum),

**Wilcoxon test

## DISCUSSION

Cardiovascular diseases, especially aortic valve diseases such as aortic stenosis, are leading causes of death globally, significantly affecting elderly individuals above the age of 65 years^[1,2,18]^. With advancements in healthcare technology and improved living conditions, the presentation of these diseases has evolved, underscoring the importance of early and accurate diagnosis and treatment^[1,3,19]^.

Severe aortic stenosis treatment is predominantly surgical, with well-established guidelines advocating for aortic valve replacement using either biological or mechanical prostheses^[3,4,5]^. Emerging studies suggest shifting surgical indications towards intermediate risk, less symptomatic patients, to further improve survival rates and reduce morbidity and mortality associated with invasive procedures.

Sutureless prostheses, like PERCEVAL®, are gaining attention for their efficacy in combined procedures and reoperations, aiming to reduce overall surgical time^[10,11]^. There are several surgical access options, including total sternotomy, J sternotomy (ministernotomy), and right lateral thoracotomy, each impacting outcomes such as hospitalization time and recovery rate^[9,20]^.

This study focuses on evaluating CPB and cross-clamping times during aortic valve replacement surgery accessed through ministernotomy, comparing conventional biological prostheses with sutureless PERCEVAL®. These intrinsic factors significantly influence surgical outcomes, such as blood loss, risk of stroke, mechanical ventilation time, and hospital stay, affecting the initial 30-day postoperative outcomes^[10,11,21]^.

Surgical techniques varied between the two study groups. Aortotomy depended on the prosthesis type, with distinct incision techniques and anchoring methods for conventional and sutureless prostheses, aiming to prevent complications^[21]^. Sutureless prostheses have documented benefits in improving patient hemodynamics by significantly reducing gradients and enhancing transvalvular flow^[18]^.

To evaluate the two groups effectively, understanding the patients’ epidemiological and clinical profile and establishing vital variables were essential. The comparison included echocardiography parameters in pre and postoperative periods, assessing cardiac sufficiency through left ventricular ejection fraction, and myocardial remodeling through left ventricular mass.

While this retrospective study offers insights, the absence of controlled, randomized clinical trials limits the full understanding of the efficacy of these prostheses through minimally invasive accesses, leaving them unrecommended in international and Brazilian guidelines^[22,23]^.

In this study on aortic valve diseases, especially aortic stenosis, we observed a demographic primarily consisting of males (61.3%), with a median age of 61 years, highlighting a similarity to the population studied by Guner et al.^[18]^. A significant age difference was noted between the Conventional Bioprostheses and PERCEVAL® groups (*P*=0.003), although both fell within the elderly bracket, forming a homogenous population for comparison.

Our study population exhibited significant tendencies toward being overweight, with around 20% categorized as obese, which aligns with findings of Guner et al.^[18]^ of patients predominantly above the normal weight range. Comorbidities such as dyslipidemia (34.1%) and diabetes mellitus (25.3%) were common, increasing the risk of cardiovascular diseases. Mujtaba et al.^[21]^ found a similar prevalence of diabetes mellitus in their study, without evaluating the presence of obesity or dyslipidemia. We observed no statistically significant difference between the two prosthesis groups concerning these comorbidities (*P*>0.05).

Hypertension was prevalent in 80.2% of our population, correlating with left ventricular hypertrophy, cardiac remodeling, and heart failure, while 12.1% were smokers, and 3.3% were alcohol consumers — extrinsic factors contributing to the development of high-risk calcific aortic stenosis. The PERCEVAL® group recorded 91.7% hypertensive patients, while the conventional bioprostheses group recorded 78.5%, indicating homogeneity. Comparatively, an English study by Mujtaba et al.^[21]^ found approximately 71% hypertensive and 63% smoking patients.

Chronic kidney disease, found in 3.3% of our population, significantly elevates perioperative morbidity and mortality, aligning with international literature indicating around 1% prevalence. No significant statistical difference was observed between the PERCEVAL® and conventional bioprostheses groups in this regard (*P*=1.000).

Additionally, 12.1% had pre-existing arrhythmias, and 6.6% had atrial fibrillation, conditions that escalate perioperative morbidity and mortality due to the dependence on pacemakers or rhythm-controlling medications, potentially facilitating thrombosis and embolic events. These conditions were not significantly different between the two groups (*P*=0.635 and *P*=0.583, respectively), compared to 15% of atrial fibrillation and 0.5% of other arrhythmias in the study by Mujtaba et al.^[21]^. This detailed demographic and clinical profile underscores the importance of understanding the patient population to explore better alternatives and solutions for aortic valve diseases, highlighting the necessity for further research into the connections between valve diseases and comorbidities. The preoperative stroke rate in our population was 4.4%, and the prevalence of CAD was 20.9%. These conditions significantly increase surgical risks for patients. When examined separately, the PERCEVAL® group showed a 41.7% prevalence of CAD, while the conventional bioprostheses group had a prevalence of 5.1% for stroke and 17.7% for CAD. However, no statistically significant differences were observed, with *P*-values of 1.000 for stroke and 0.119 for CAD, both > 0.05. Nevertheless, when compared with another international study by Guner et al.^[18]^, it was noted that 3% had preoperative stroke, and 8% had CAD.

It is evident that as the number of comorbidities increases, surgical risks rise, potentially leading to longer hospital stays, which result in higher costs for healthcare institutions^[8,24]^.

An advantage of our study is that all patients were operated on by the same team, with the same surgeon, who has already achieved a high level of technical excellence.

CPB and cross-clamping times are two variables that correlate with postoperative negative outcomes. In the conventional bioprostheses group, the median CPB time was 61 (55-70) minutes, and the cross-clamping time was 41 (37-49) minutes. In comparison, the PERCEVAL® group had a median CPB time of 59.5 (45-62.8) minutes and cross-clamping time of 39.5 (28-39.5) minutes. In an English study by Mujtaba et al.^[21]^, the authors found a median cross-clamping time of 37 minutes for the PERCEVAL® group and 52 minutes for the conventional bioprostheses group. Regarding CPB time, they reported 59 minutes for the PERCEVAL® group and 76 minutes for the bioprostheses group. Similarly, Santarpino et al.^[23]^ observed a median CPB time of 65 minutes and cross-clamping time of 48 minutes for sutureless valve implantation through ministernotomy, while conventional prostheses implantation through total sternotomy had a median CPB time of 73 minutes and cross-clamping time of 58 minutes. These findings indicate a statistically significant difference in cross-clamping time (*P*=0.0139). CPB time directly affects postoperative results, potentially reducing costs and improving postoperative conditions for the studied patients^[24]^.

Several important factors that may influence the outcomes of aortic valve replacement with sutureless or conventional bioprostheses are linked to the following aspects: length of hospital stay, which directly impacts healthcare costs, appropriate selection of the prosthesis, correct sizing based on the patient's body surface area, etiology of the valve disease, which may indicate possible postoperative complications, and intraoperative diagnosis, which provides insight into the patient's real risk during aortic valve replacement. No statistically significant differences were found between the two groups.

Descriptively, the length of stay in the intensive care unit was two days for both groups, while the median hospital stay was four times longer (eight days). It is important to note that this was a multicenter study, and procedures were performed in different hospitals with varying postoperative teams and institutional protocols, potentially contributing to variations in the length of stay. A study involving nine Italian cardiac surgery centers showed that the hospital stay duration, related to the effectiveness of sutureless prostheses and their cost-effectiveness, led to an increase of EUR 479.45 in total hospitalization cost. The cost-effectiveness for sutureless prostheses implantation through ministernotomy was EUR 24,181.5^[24]^. This raises questions about whether it is better to invest in a more expensive yet more effective prosthesis, or to save on costs initially, potentially incurring higher costs through readmissions and reoperations over time.

Regarding early outcome variables and mortality in the first 30 post-procedure days, there were no instances of MI or cardiac tamponade. Bleeding, defined by Bojar 2021, was based on the amount of blood extravasated in the tubes: > 1.5 mL/kg/hour for six consecutive hours, 2 mL/kg/hour for three consecutive hours, or 3 mL/kg/hour for two consecutive hours. According to the 2014 universal definition of bleeding, it is considered moderate when it ranges from 801 to 1,000 mL/12 hours, severe when it ranges from 1,001 to 2,000 mL/12 hours, and massive when it exceeds 2,000 mL/12 hours. Bleeding was observed in 2% of patients, which is consistent with the literature and is directly related to the duration of CPB, as it results in the consumption of blood cells, especially platelets, as noted by Jiritano et al.^[25]^. Additionally, in the first postoperative year, nitinol may be related to a significant drop in platelet count.

A subset of the study population required permanent pacemaker implantation, accounting for 4.4% of cases. This is directly related to the etiology of the disease and how the valve prosthesis is anchored to the aortic ring, potentially affecting the conduction system. An English study observed a higher rate of pacemaker implantation in the conventional bioprostheses group (12%) compared to the PERCEVAL® group (5%), which can reduce the costs associated with implantable devices in the postoperative period^[21]^.

In our study, postoperative strokes were infrequent, occurring in only 1.1% of patients within the studied groups. This risk is more prominent in the conventional bioprostheses group due to the potential displacement of calcium plaques during the tightening of fixation knots. Most patients were diagnosed with aortic valve calcification (78%), with significant aortic valve stenosis (74.7%), placing them in the moderate risk category for symptomatic calcified aortic lesions. Furthermore, despite the high prevalence of rheumatic valvular disease, most patients presented with calcific disease. Only 16.5% of patients in the conventional bioprostheses group and 14.3% in the PERCEVAL® group had rheumatic aortic valve disease, highlighting socioeconomic valuations within the study population.

In accordance with the study's goals, comparative evaluations of transthoracic and transesophageal echocardiography parameters were performed in all patients before and after surgery. This aimed to assess hemodynamic improvements, enhanced cardiac capacitance, and progress toward better cardiac sufficiency between the two prosthesis groups. A statistically significant difference was observed in transthoracic echocardiogram parameters between the preoperative and postoperative periods for all patients. Specifically, left ventricular ejection fraction, left ventricular diastolic diameter, left ventricular mass, and maximum and mean systolic gradients showed higher medians in the preoperative period. While left ventricular ejection fraction slightly decreased, it remained > 45%, as per valvular heart disease guidelines^[22]^, thus preserved. Increased left diastolic diameter and left ventricular mass indicate advanced aortic valvular disease, which is apparent in the preoperative period and subsequently reduced in the postoperative period after removal of the diseased valve's resistance. In the postoperative period, there was a reduction in diastolic diameter due to myocardial remodeling following removal of valve resistance, along with a decrease in eccentric hypertrophy of the posterior wall and interventricular septum, leading to a significant drop in maximum and mean gradients (*P*<0.01).

An important distinction of our study lies in its comprehensive comparison of pre and postoperative echocardiograms across the entire patient cohort, a practice not commonly observed in most other studies. It is important to note that although echocardiograms were performed following the American Society of Echocardiography definitions, the exam's interpretation is operator-dependent and may have important variations. Moreover, immediate postoperative bedside assessment is suboptimal because the myocardium is still recovering and seeking homeostatic balance following cardioplegic arrest and specific structural architectural changes.

Additionally, a comparative evaluation of echocardiographic parameters between the two prosthesis groups (conventional bioprostheses *vs.* PERCEVAL®) in both pre and postoperative periods showed no statistically significant differences. Descriptively, notable differences were observed in the PERCEVAL® group, particularly a greater reduction in maximum and mean systolic gradients in both pre and postoperative periods, indicating a larger hemodynamic gain and an increased effective orifice area. This finding translates to a more immediate benefit for patients who underwent PERCEVAL® implantation. Similar hemodynamic gains, especially in patients with small aortic annuli, have been reported in other studies^[18,26]^. The comparable performance of sutureless valve replacement surgery underscores the feasibility of this technique. However, controlled, randomized clinical trials with robust sample sizes are needed to establish better comparisons and introduce new therapeutic devices into international surgical recommendations for physicians.

### Limitations

Our study was limited by the relatively small sample size, as several variables influenced the quantity. Firstly, PERCEVAL® is not an available option in the public healthcare system. The sutureless prostheses used in this study were implanted in a specific group with private medical assistance (a total of 12 patients). Another variable that limited the number of patients was aortic valve replacement with conventional bioprostheses through a minimally invasive ministernotomy approach, which requires an experienced and skilled surgical team. Lastly, the analysis was conducted for primary surgeries and not for combined procedures. Thus, the importance of sutureless prostheses as an alternative for aortic valve replacement via ministernotomy is evident, as they can reduce hospital costs while achieving positive outcomes.

## CONCLUSION

According to this study, we could observe that the sutureless PERCEVAL® prosthesis emerges as a viable alternative and provides similar hemodynamic gains and clinical results. Additionally, the ministernotomy access route enhances recovery, offering greater benefits to patients. Therefore, techniques aimed at reducing CPB and cross-clamping times are potentially more beneficial for clinical outcomes and increased survival, reducing the number of complications related to cardiac surgery.

Randomized studies need to be conducted to introduce the use of sutureless prostheses with stronger levels of evidence into valvular disease guidelines, thereby reducing costs related to both the prosthesis itself and hospitalization while increasing the benefits of outcomes achieved for patients. Finally, bringing sutureless prostheses into the Brazilian public healthcare system could be a viable alternative as new studies are compiled and demonstrate positive results, ultimately reducing long-term adverse consequences for patients with aortic valve diseases.

## Abbreviations, Acronyms & Symbols

AVR: Aortic valve replacement
ICFs: Informed Consent Form
CAD: Coronary artery disease
InCor-PE: Instituto do Coração de Pernambuco
CPB: Cardiopulmonary bypass
MI: Myocardial infarction
